# A New Twist on an Old Technique: Lawson Retrograde Endoscopic-Guided Nephrostomy Access for Percutaneous Nephrolithotomy in Prone Split-Leg Position

**DOI:** 10.1089/cren.2018.0073

**Published:** 2018-11-29

**Authors:** Kamaljot S. Kaler, Egor Parkhomenko, Cyrus Y. Lin, Zachary A. Valley, William K. Kim, Zhamshid Okhunov, Roshan M. Patel, Jaime Landman

**Affiliations:** ^1^ Department of Urology, University of California, Irvine, California.; ^2^ Department of Surgery, Section of Urology, University of Calgary, Calgary, Alberta, Canada.

**Keywords:** retrograde nephrostomy, Lawson catheter, percutaneous nephrolithotomy, nephrolithiasis, renal calculi, percutaneous nephrostomy

## Abstract

***Background:*** A minority of urologists performing percutaneous nephrolithotomy (PCNL) achieve their own nephrostomy access. In an effort to simplify the access part of PCNL, we herein describe our initial experience with endoscopic-guided retrograde percutaneous access in the prone split-leg position.

***Case Presentation(s):*** After informed consent, a confirmed negative urine culture, and 1 week pretreatment with tamsulosin, four carefully selected PCNL patients underwent endoscopic-guided retrograde access in a prone split-leg position using the Lawson catheter. In all the four patients, we achieved endoscopic-guided retrograde upper pole access in the prone split-leg position. A single Clavien 3B complication occurred. Total fluoroscopy time for the PCNL averaged 162 seconds (51–283). Complete stone-free rate at 1 week based on CT scan was 25%, and a stone-free rate defined as <4 mm was 100%.

***Conclusion:*** Endoscopic-guided retrograde percutaneous upper pole access can be established efficiently with a modified Lawson technique in the prone split-leg position.

## Introduction and Background

Achieving percutaneous access is the crux of a percutaneous nephrolithotomy (PCNL). Access is most often achieved using fluoroscopy and/or ultrasound guidance through an antegrade needle-guided approach. However, this technique is often associated with a steep learning curve, requiring >60 cases before the surgeon becomes competent and >100 cases to gain proficiency.^1^ An alternative approach for access is using a retrograde approach as initially described by Lawson in 1984.^2^ This technique was not widely accepted because of concerns over lack of control of the Lawson needle (i.e., “rocket” wire) as it exited the kidney and was advanced toward the skin of the flank. Improvements in PCNL technology, such as the routine use of the ureteroscope, ureteral access sheath, and modified lithotomy position, caused us to rethink the Lawson retrograde technique from an endoscopically guided standpoint. In our series we further modified this technique to include access in the prone split-leg position.

## Presentation of Cases

### Patient selection and preoperative planning

**FIG. 1.**
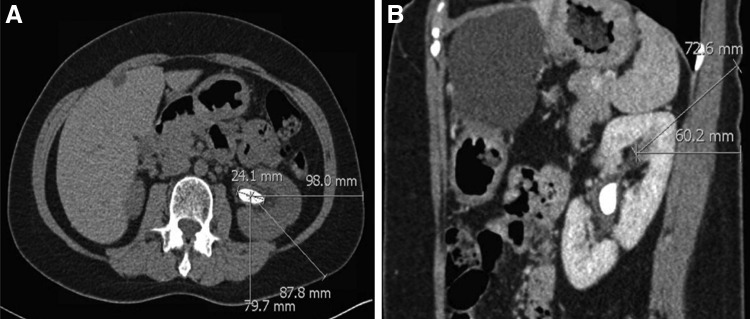
CT imaging illustrating renal calculi with skin-to-stone distance. **(A)** Axial view measuring 79.7 mm. **(B)** Sagittal view measuring 72.6 mm.

Prospective PCNL patients were selected for the retrograde access approach after careful review of their preoperative low-dose stone protocol CT scan, which included axial, coronal, and sagittal views. After assessment of tract length and the anatomy of the collecting system (i.e., ease of access to the stone through an upper pole approach), we chose patients with a potential nephrostomy tract length ≤10 cm on sagittal CT imaging whose access to the upper pole calix would also be subcostal (Fig. 1).

Three men and one woman, all with a documented preoperative sterile urine culture, were selected: average age 60.5 years old (55–65), body mass index (BMI) 28.1 kg/m^2^ (19.5–34.7), and mean American Society of Anesthesiologists score 2.5 (2–3) (Table 1). Mean stone cumulative diameter was 2.5 cm (1.5–3.3). Mean skin-to-stone distance was 9.61 cm (8.5–10.6); preoperative presumed nephrostomy tract length was 6.8 cm (5.8–8.5).

**Table 1.** Patient, Stone, and Surgical Characteristics

*Variable*	
No. of patients	4 (3 men, 1 woman)
Age in years (mean)	60.5 (55–65)
BMI (mean)	28.1 (19.5–34.7)
ASA (mean)	2.5 (2–3)
Mean skin-to-stone distance (cm)	9.61 (8.5–10.6)
Mean number of tracts	1
Mean tract length (cm)	6.8 (5.8–8.5)
Mean preoperative cumulative stone diameter (cm)	2.5 (1.5–3.3)
Mean preoperative stone volume (mm^3^)	1840 (924–3038)
Mean absolute stone volume (mm^3^) reduction (%)	1826 (99)
Mean case time (minutes)	183 (143–246)
Mean fluoroscopy time for case (seconds)	162.3 (51–283)
Mean hospital stay (days)	1.75 (1–4)
Mean preoperative hematocrit (%)	42.6 (36.7–45.5)
Mean postoperative hematocrit (%)	39.65 (36.7–42.1)
Ureteral injury mean (PULS)	0.75 (0–2)
Stone-free rate (%) on CT scan postoperative day 1: complete stone-free rate	25
<4 mm stones	100
Mean preoperative HU	872 (400–1111)
Postoperative ED visits or readmission	0
Concomitant procedures	Contralateral ureteroscopy (*n* = 3), urethral stricture dilation (*n* = 1)
Complications (*n* = 1)	Clavien IIIb (stent exchange)

ASA = American Society of Anesthesiologists; BMI = body mass index; ED = emergency department; PCNL = percutaneous nephrolithotomy; PULS = postureteroscopic lesion scale.

After obtaining informed consent, each patient was pretreated with oral tamsulosin 0.4 mg once daily for 7 days to possibly facilitate ureteral relaxation before access sheath placement during surgery. Patients were also given oral ciprofloxacin 500 mg twice daily (or alternative antibiotic if allergic to ciprofloxacin) for 7 days before surgery, per standard of care for all large volume stone ablative surgeries at our institution.

### Technique

#### Patient positioning and ureteroscopy

The patient positioning and ureteroscopy were performed as previously described.^3^

#### Endoscopic-guided retrograde Lawson access through an upper pole posterior calix

##### Lawson nephrostomy catheter preparation

**FIG. 2.**
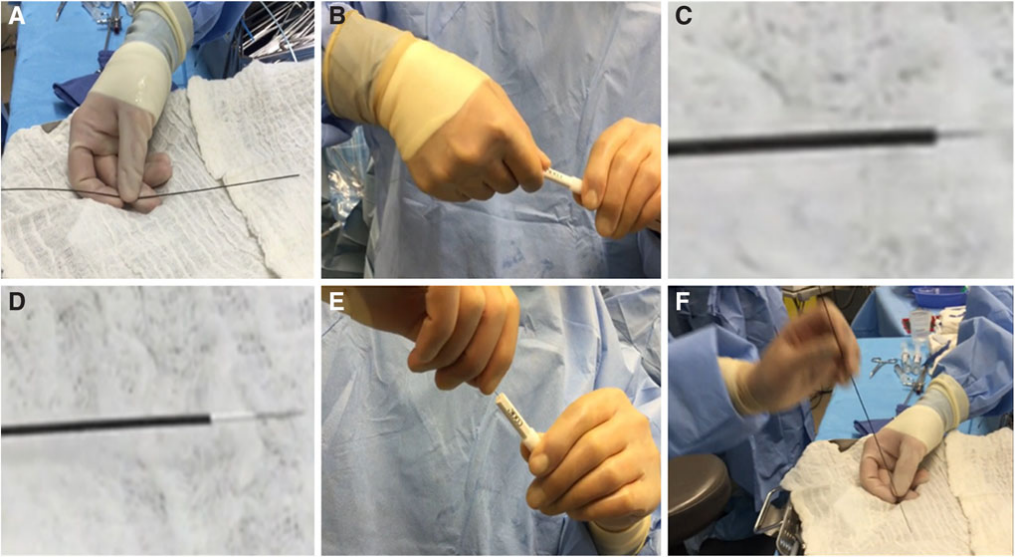
Lawson retrograde needle-catheter preparation. **(A, B)** Luer lock loosened. **(C, D)** Needle advanced and secured inside sheath. **(E, F)** Luer lock secured with needle just inside sheath to prevent damage to ureteroscope.

The Luer lock on the catheter was loosened to advance the needle such that it was positioned just inside the device's sheath. The Luer lock was then tightened and the Lawson catheter assembly was advanced until it resided just inside the distal end of the working port (Fig. 2).

##### Ureteroscopy and retrograde access

**FIG. 3.**
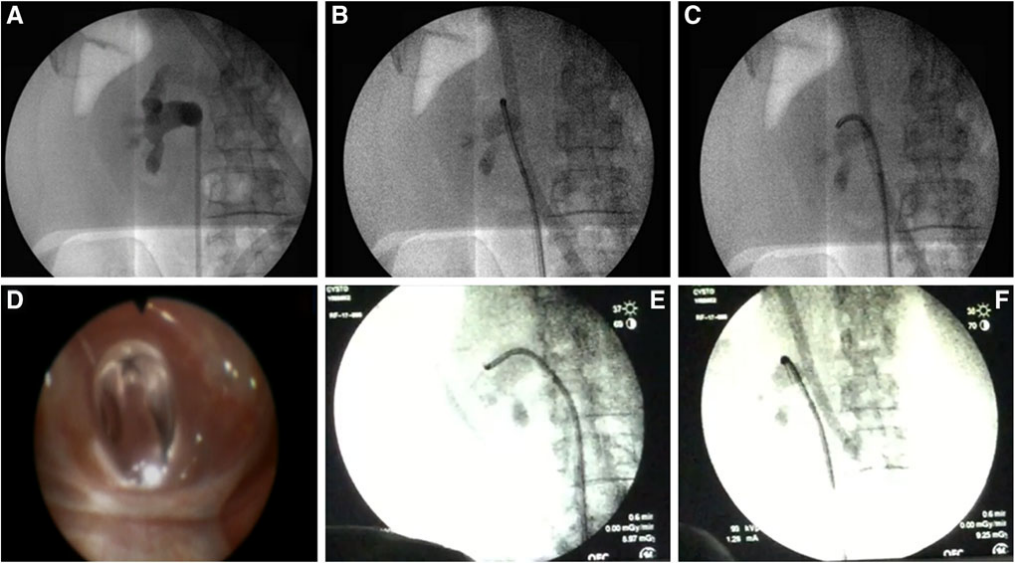
Lawson retrograde endoscopic-assisted access. **(A)** Retrograde pyelogram. **(B, C)** Identifying posterior upper pole calix. **(D–F)** Posterior calix and Lawson rocket wire exiting ureteroscope.

**FIG. 4.**
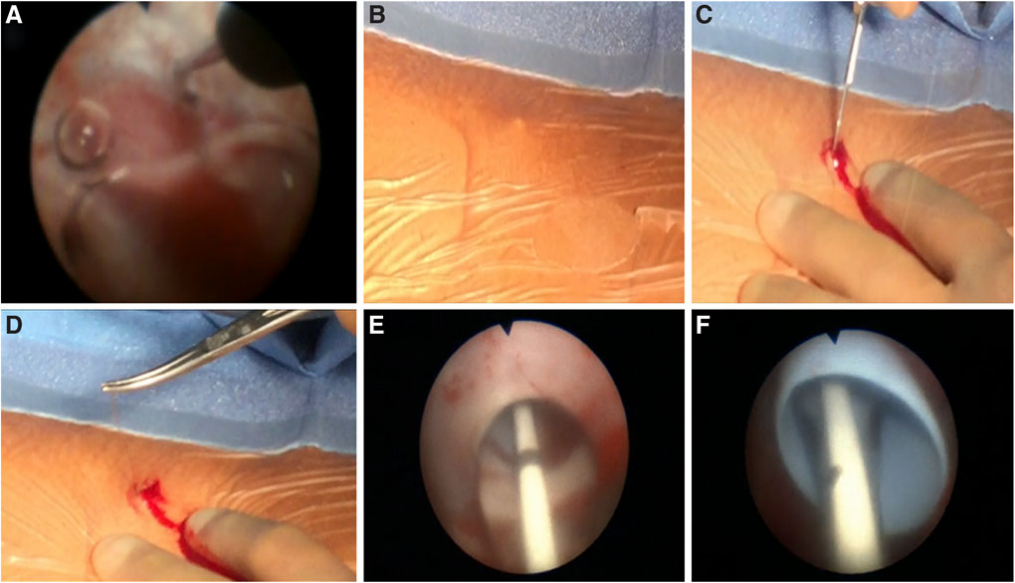
Lawson retrograde endoscopic-assisted access. **(A)** Needle advancement through the ureteroscope. **(B–D)** External view of patient's flank with wire exiting. **(E, F)** Endoscopic view of balloon dilation and nephrostomy sheath advancement.

Using a 10 cc syringe, 2 cc of air was slowly injected through the ureteroscope. The ureteroscope was moved into a posterior (i.e., air-containing) upper pole calix; this was also confirmed fluoroscopically (Fig. 3). The Luer lock on the Lawson needle was loosened. With respirations suspended by the anesthesiologist, the needle was advanced out of the ureteroscope into a posterior upper pole fornix. Using an oblique fluoroscopic view, we tracked the needle to confirm that its path was straight and posterior (Fig. 3). As the needle encountered the skin, tenting of the skin was noted and an incision was made over the tip of the Lawson needle, which was then grasped and retrieved from the flank (Fig. 4). The incision was extended to 1 cm, and a 5 mm fascial incising needle was advanced over the Lawson wire followed by passage of an 8F dilator over the Lawson wire; as the ureteroscope was removed, the 8F catheter was advanced further until it rested in the ureteral access sheath. The ureteroscope and the Lawson wire were exchanged for an antegrade 260 cm exchange guidewire thereby establishing through-and-through access from the urethral meatus to the flank. The ureteroscope was reinserted into the 16F access sheath alongside the exchange guidewire to observe entry of the 10 mm nephrostomy balloon (NephroMax or X-Force) into the calix. The balloon was inflated to 30F under ureteroscopic vision. The 30F Amplatz access sheath was positioned in the collecting system also under ureteroscopic control. The 10 mm nephrostomy balloon was deflated and removed; lithotripsy was begun with the 1000 μm holmium laser through the rigid nephroscope.^3^

#### Lithotripsy, stone fragment extraction, stent insertion, and sealing of nephrostomy access

Lithotripsy was performed as previously described.^3^

#### Operative results

We achieved retrograde access in the prone-split leg position through an upper pole posterior subcostal calix in all the four patients with a single pass of the Lawson wire (Table 1). The mean operative time was 183 minutes (143–246) and the mean fluoroscopy time was 162 seconds (51–283). Mean tract length was 6.8 cm (5.8–8.5). A CT scan at postoperative day 1 revealed stone-free rate of 25%; significant fragment-free rate (i.e., <4 mm) was 100%. Postureteroscopic lesion scale score upon removal of the 16F ureteral access sheath was a mean of 0.75 (0–2). There was one complication (Clavien 3B). This patient had a postoperative bleeding episode resulting in clot obstruction of the stent and a urinoma formation; this patient required stent exchange but no transfusion or angiography. The patient's condition improved immediately after stent exchange; the hematocrit fell minimally (i.e., from 43.5% preoperative to 40.1% postoperative).

## Discussion and Literature Review

PCNL has significantly benefited from technological advances. In 1975, the first documented PCNL case used antegrade fluoroscopy alone to guide stone removal. Nearly 50 years later, urologists continue to primarily use the same antegrade fluoroscopy approach either alone or in combination with ultrasound to establish access; also, ureteroscopy is used by some urologists to help guide antegrade access.

In our series, we modified the ureteroscopic Lawson technique of retrograde access by using it in the prone position for upper pole access under ureteroscopic guidance. One theoretical advantage of this approach is the ability to accurately target the fornix while holding the needle in a stable position such that the needle is more likely to be advanced directly posteriorly, thereby providing a straighter and shorter nephrostomy tract compared with the traditional retrograde access in modified supine. In addition, if retrograde access failed, one could transition to the traditional endoscopic-guided fluoroscopic or ultrasound-monitored antegrade access since the patient is already prone. Of note, an injection of a small amount of air through the ureteroscope invariably settles in the most posterior upper pole calix thereby facilitating selection of the most appropriate calix for puncture, which is not the case when the patient is supine. Also by doing this under endoscopic control, the surgeon can carefully select the site of puncture insuring that the needle traverses either the fornix or the papilla directly. We have previously described a method of antegrade puncture access, which is facilitated by endoscopic vision; however, we believe a slight improvement to this technique is warranted given that the Lawson retrograde method may reduce fluoroscopy exposure, decrease access time, and increase the accuracy of access.

We recognize that the antegrade bullseye method for percutaneous access is a time-tested standard technique; however, rarely is a true bullseye achieved when one tries to puncture the targeted calix by passing a needle from the flank to the caliceal fornix. Often the target is narrowly missed thus potentially contributing to associated complications, such as hemorrhage or difficult access to the stone itself. Instead, targeting the caliceal fornix in a retrograde manner has the ability to reduce radiation exposure while also making the tract more precise as the fornix is punctured initially and under direct vision as the rocket wire is pushed outward toward the flank. In addition, a potential benefit of using the retrograde approach for access is the shortened learning curve. The traditional antegrade PCNL is historically difficult, which often requires the expertise of an interventional radiologist. In contrast, the retrograde access with the Lawson technique requires only 10 to 20 procedures, as opposed to 100 cases, to become proficient.^1^ This is promising for the practicing urologist as a vast majority (80%) of PCNL accesses in the United States are gained by interventional radiologists.^3^ Furthermore, El-Assmy and colleagues examined surgical records of 1121 patients who underwent PCNL through urologist or radiologist established access, and found that patients with access achieved by the urologist had a significantly higher volume of complex and challenging cases, and yet the complication and stone-free rates were similar to those simpler cases in which access was achieved by the interventional radiologist.^4^

There are definite limitations to our report. First, as with all initial reports of a technique, our population size is small with only four patients. A larger cohort is necessary to better test the safety and feasibility of the approach. Second, at our institution, all of our PCNL cases are done with ureteroscopic assistance through an ureteral access sheath to improve accuracy of needle, balloon dilator, and sheath placement, facilitate stone clearance, and maintain a low-pressure environment during PCNL. We recognize that this may add costs to the procedure caused by the expense of the ureteral access sheath (i.e., $113.55 USD); however, we believe that this expense is justified as it allows for the lowest intrarenal pressures while also allowing stone fragments to be evacuated rapidly through the access sheath. Third, a large renal pelvis stone burden may require the surgeon to laser a channel through the stone to access the posterior upper pole calix; this would most certainly increase the overall time to acquire access. Furthermore, we limited the technique to patients who were not overly obese and in whom the approach would be subcostal; higher BMI (>40) patients commonly require >10 cm nephrostomy tracts. Indeed, BMI has been reported to be the most predictive indicator of failure of retrograde access. Finally, using this approach for a supracostal access is concerning given the placement of the liver, spleen, and pleura as well as the potential for interference from an overlying rib.

## Conclusion

An endoscopic-guided retrograde percutaneous nephrostomy tract was established with the Lawson needle wire in four consecutive patients in a prone split-leg position.

## Abbreviations Used

BMI: body mass index
CT: computed tomography
ED: emergency department
HU: Hounsfield units
PCNL: percutaneous nephrolithotomy
PULS: postureteroscopic lesion scale
